# Supplementary material for: Structure and Photocatalytic Properties of Ni-, Co-, Cu-, and Fe-Doped TiO2 Aerogels
Source: Gels. 2023 Apr 24;9(5):357. doi: 10.3390/gels9050357 (PMC10217561; doi:10.3390/gels9050357)
Supplement: Supplementary file 1 [file gels-09-00357-s001.zip › gels-2281523-supplementary.pdf]

Supplementary Materials

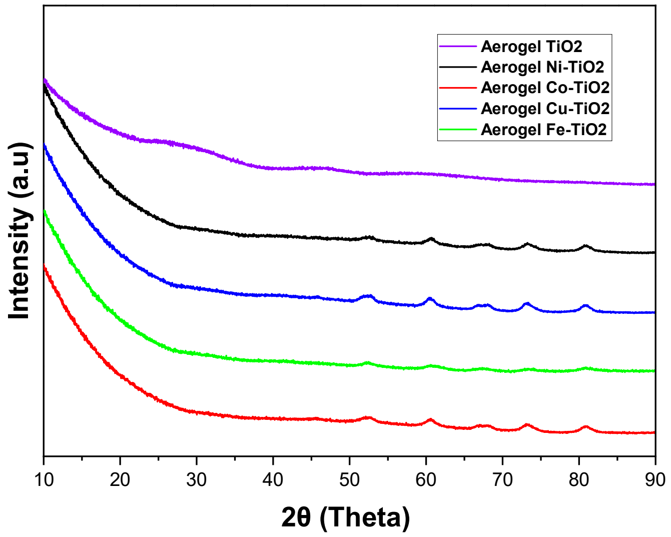

Figure S1. XRD spectra of the metal-doped TiO_2 aerogels after supercritical drying process.

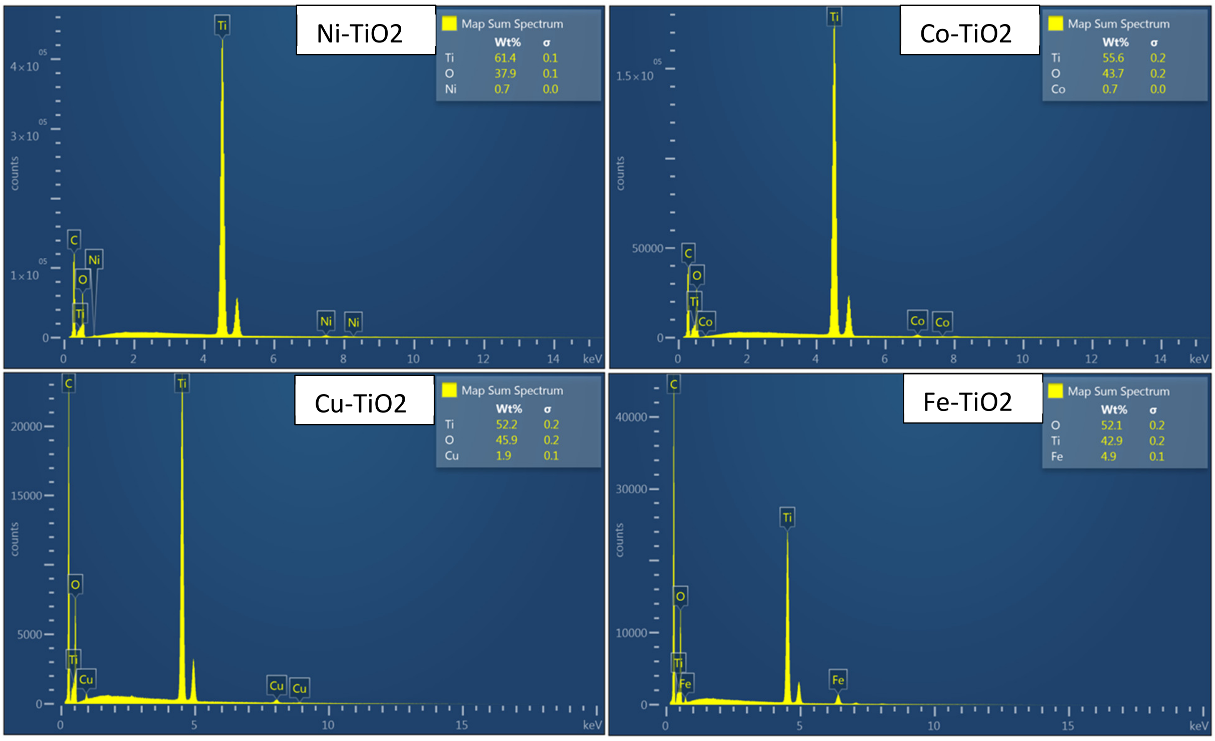

Figure S2. EDS spectra of the metal-doped TiO_2 aerogels after calcined at 500 °C.

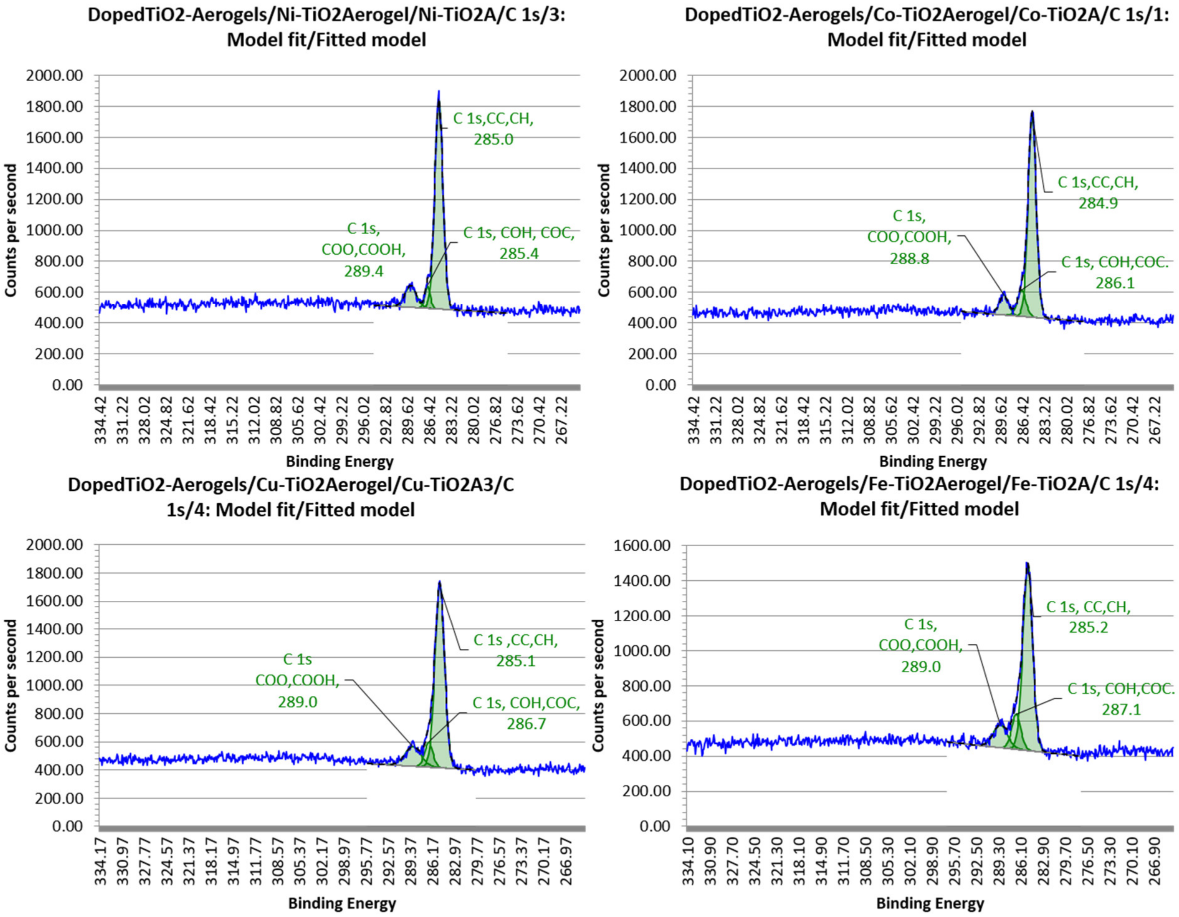

Figure S3. Deconvoluted binding energies and atomic concentrations of C 1s obtained from the primary XPS spectra from metal-doped TiO_2 aerogels calcined at 500 °C.

Table S1. Chemical quantification from the wide XPS spectra of the metal-doped TiO_2 aerogels.

Quantification

**Ni-TiO2**	BE [eV]	FWHM [eV]	RSF	Atomic conc. [%]	Error [%]	Mass conc. [%]	Error [%]
C 1s 285.5	285.09	2.42	0.28	13.02	0.96	6.45	0.51
Ti 2p 459.4	464.57	2.54	2.00	8.94	0.33	17.65	0.58
Ti 2p 458.4	459.00	1.73	2.00	17.85	0.37	35.24	0.59
O 1s 530.4	530.30	1.93	0.78	59.66	0.82	39.37	0.63
Ni 2p 856.4	856.34	1.50	4.04	0.16	0.09	0.39	0.21
Ni 2p 861.4	862.60	4.55	4.04	0.37	0.26	0.90	0.63
**Co-TiO2**	BE [eV]	FWHM [eV]	RSF	Atomic conc. [%]	Error [%]	Mass conc. [%]	Error [%]
C 1s 285.9	284.96	2.19	0.28	11.95	0.95	5.78	0.49
Ti 2p 459.2	458.87	1.78	2.00	18.54	0.39	35.76	0.62
Ti 2p 464.2	464.50	2.80	2.00	9.96	0.38	19.22	0.64
O 1s 530.2	530.18	1.91	0.78	59.07	0.84	38.09	0.65
Co 2p 782.2	781.93	2.38	3.59	0.29	0.17	0.69	0.41
Co 2p 787.2	788.62	3.58	3.59	0.20	0.26	0.47	0.61
**Cu-TiO2**	BE [eV]	FWHM [eV]	RSF	Atomic conc. [%]	Error [%]	Mass conc. [%]	Error [%]
C 1s 284.9	284.91	2.53	0.28	19.72	1.11	10.31	0.65
Ti 2p 458.9	459.48	2.53	2.00	15.15	0.43	31.58	0.72
Ti 2p 459.9	465.02	3.31	2.00	7.99	0.39	16.66	0.72
O 1s 530.9	531.01	3.11	0.78	56.34	1.02	39.24	0.82
Cu 2p 933.9	934.30	3.37	5.32	0.62	0.15	1.71	0.41
Cu 2p 955.9	954.46	4.66	5.32	0.18	0.20	0.50	0.56
**Fe-TiO2**	BE [eV]	FWHM [eV]	RSF	Atomic conc. [%]	Error [%]	Mass conc. [%]	Error [%]
C 1s 284.3	284.42	2.44	0.28	13.89	1.12	6.95	0.61
Ti 2p 458.3	458.21	2.09	2.00	15.47	0.43	30.84	0.82
Ti 2p 463.3	463.73	3.12	2.00	8.57	0.41	17.08	0.77
O 1s 529.3	529.59	2.41	0.78	59.81	1.07	39.86	0.98
Fe 2p 711.3	711.54	3.37	2.96	1.09	0.33	2.53	0.76
Fe 2p 724.3	724.83	6.36	2.96	1.18	0.63	2.74	1.44

Table S2. Quantification of deconvoluted binding energies and atomic concentrations of C 1s obtained from the primary XPS spectra from metal-doped TiO_2 aerogels calcined at 500 °C.

Quantification

$Ni-TiO_2$	BE [eV]	FWHM [eV]	RSF	Atomic conc. [%]	Error [%]	Mass conc. [%]	Error [%]
C 1s,CC,CH, 285.0	284.97	1.38	0.28	82.08	3.76	82.08	3.76
C 1s, COO,COOH, 289.4	289.09	1.94	0.28	12.17	3.61	12.17	3.61
C 1s, COH, COC, 285.4	286.62	0.88	0.28	5.75	1.84	5.75	1.84
Quantification							
$Fe-TiO_2$	BE [eV]	FWHM [eV]	RSF	Atomic conc. [%]	Error [%]	Mass conc. [%]	Error [%]
C 1s, CC,CH, 285.2	285.06	1.60	0.28	73.55	4.34	73.55	4.34
C 1s, COH,COC. 287.1	286.71	1.52	0.28	13.16	2.93	13.16	2.93
C 1s, COO,COOH, 289.0	288.99	2.34	0.28	13.30	4.14	13.30	4.14
Quantification							
$Co-TiO_2$	BE [eV]	FWHM [eV]	RSF	Atomic conc. [%]	Error [%]	Mass conc. [%]	Error [%]
C 1s,CC,CH, 284.9	284.98	1.35	0.28	79.93	3.64	79.93	3.64
C 1s, COH,COC. 286.1	286.53	1.42	0.28	10.86	2.75	10.86	2.75
C 1s, COO,COOH, 288.8	289.12	1.57	0.28	9.21	2.99	9.21	2.99
Quantification							
$Cu-TiO_2$	BE [eV]	FWHM [eV]	RSF	Atomic conc. [%]	Error [%]	Mass conc. [%]	Error [%]
C 1s ,CC,CH, 285.1	285.08	1.58	0.28	77.13	4.19	77.13	4.19
C 1s, COH,COC, 286.7	286.66	1.36	0.28	8.92	2.40	8.92	2.40
C 1s COO,COOH, 289.0	288.95	2.68	0.28	13.95	4.05	13.95	4.05

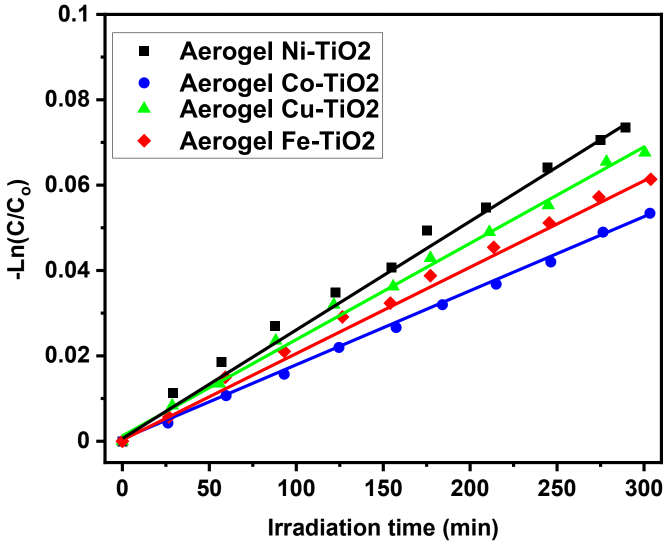

Figure S4. Photocatalytic performance of the metal-doped TiO_2 aerogels: Ni, Co, Cu, Fe fitting the kinetic reaction curves -ln(C/Co)= $k_{app}t$, where k_{app} refers to the apparent rate of photoactivity for the samples calcined at 900°C.

Table S3. STEM-EDS elemental quantification of the Ni, Ti, and O presented in the Ni-TiO2 aerogel lattice after calcined at 500°C.

Z	Element	Family	Atomic Fraction (%)	Atomic Error (%)	Mass Fraction (%)	Mass Error (%)	Fit error (%)
8	O	K	62.85	8.08	36.07	3.04	0.79
22	Ti	K	36.77	6.79	63.14	9.91	0.03
28	Ni	K	0.38	0.07	0.8	0.13	0.64
